# Temporary Enlargement

**Published:** 1890-10

**Authors:** 


### TEMPORARY ENLARGEMENT.

We have found it necessary to increase this number sixteen pages, or one form. The enlargement will be confined to this issue, and has been rendered necessary by the accumulation of much valuable material. It is to be hoped that our contributors will appreciate this as an evidence of the desire of the management to present their articles at as early a date as possible.

